# Severe infection incidence among young infants in Dhaka, Bangladesh: an observational cohort study

**DOI:** 10.1136/bmjph-2024-002383

**Published:** 2025-07-13

**Authors:** Alastair Fung, Cole Heasley, Lisa G Pell, Diego G Bassani, Prakesh S Shah, Shaun K Morris, Davidson Hamer, Mohammad Shahidul Islam, Abdullah Al Mahmud, Eleanor Pullenayegum, Samir K Saha, Rashidul Haque, Md Iqbal Hossain, Chun-Yuan Chen, Abby Emdin, Karen M O’Callaghan, Miranda G Loutet, Shamima Sultana, S M Masum Billah, S M Abdul Gaffar, Enamul Karim, Sharika Sayed, Sultana Yeasmin, Md Mahbubul Hoque, Tahmeed Ahmed, Shafiqul A Sarker, Daniel E Roth

**Affiliations:** 1 Department of Paediatrics, The Hospital for Sick Children, Toronto, Ontario, Canada; 2 Centre for Global Child Health, The Hospital for Sick Children, Toronto, Ontario, Canada; 3 University of Toronto Dalla Lana School of Public Health, Toronto, Ontario, Canada; 4 Child Health Evaluative Sciences, The Hospital for Sick Children Research Institute, Toronto, Ontario, Canada; 5 Mount Sinai Hospital, Toronto, Ontario, Canada; 6 Department of Global Health, Boston University School of Public Health, Boston, Massachusetts, USA; 7 Section of Infectious Diseases, Boston University Chobanian & Avedisian School of Medicine, Boston, Massachusetts, USA; 8 Child Health Research Foundation, Dhaka, Bangladesh; 9 International Centre for Diarrhoeal Disease Research Bangladesh, Dhaka, Bangladesh; 10 Department of Nutritional Sciences, King’s College London, London, UK; 11 Bangladesh Shishu Hospital and Institute, Dhaka, Bangladesh

**Keywords:** Epidemiologic Research Design, Disease Transmission, Infectious, Public Health, Prevalence

## Abstract

**ABSTRACT:**

**Introduction:**

Heterogeneity in definitions of severe infection, sepsis and serious bacterial infection (SBI) in infants limits the comparability of randomised controlled trials (RCTs) of infection prevention interventions. To inform the design of infection prevention RCTs for infants in low-resource settings, we estimated the incidence of severe infection and death among Bangladeshi infants aged 0–60 days using variations in case definitions.

**Methods:**

Among 1939 infants born generally healthy in Dhaka, Bangladesh, severe infection was identified through up to 12 scheduled community health worker home visits from 0 to 60 days of age or through caregiver self-referral. The primary severe infection case definition combined physician documentation of standardised clinical signs and/or diagnosis of sepsis/SBI, plus either a positive blood culture or parenteral antibiotic treatment for ≥5 days. Incidence rates were estimated for the primary severe infection definition, the WHO definition of possible SBI, blood culture-confirmed infection and five alternative definitions including non-injury death.

**Results:**

Severe infection incidence per 1000 infant-days was 1.2 (95% CI 0.97 to 1.4) using the primary definition, 0.84 (0.69 to 1.0) using the WHO definition of possible SBI, 0.026 (0.0085 to 0.081) using blood culture-confirmed infection and 0.061 (0.029 to 0.13) for death. One-third of cases met criteria for the primary severe infection definition through physician diagnosis of sepsis/SBI rather than the standardised clinical signs, and 85% of cases were identified following caregiver self-referral despite frequent scheduled visits.

**Conclusions:**

Severe infection incidence in infants varied considerably by case definition. Using a clinical sign-based definition may miss a substantial proportion of cases identified by physician diagnosis of sepsis/SBI. A consensus definition of severe infection in infants that balances permissiveness and stringency and can be operationalised in low-resource countries would improve the comparability of RCTs. If health facilities are accessible and caregivers readily seek care for infant illness, frequently scheduled home assessments may not be necessary.

**WHAT IS ALREADY KNOWN ON THIS TOPIC**

- Randomised controlled trials (RCTs) for severe infection prevention or treatment in young infants use a diverse range of case definitions, including culture-confirmed severe infection, a combination of clinical signs and culture confirmation, and a combination of clinical signs and laboratory investigation results.
- Incidence estimates of various severe infection case definitions that can be operationalised in low and middle-income countries (LMICs) are needed to determine the feasibility of using these definitions in severe infection prevention and treatment RCTs for young infants in these settings.

**WHAT THIS STUDY ADDS**

- We demonstrate that in young infants (0–60 days of age) born generally healthy in Dhaka, Bangladesh, incidence estimates of severe infection vary considerably depending on whether a permissive or stringent case definition is adopted.
- We also demonstrate that, in this study, most severe infection cases were identified following caregiver self-referral rather than during scheduled home assessments by study personnel.

**HOW THIS STUDY MIGHT AFFECT RESEARCH, PRACTICE OR POLICY**

- Our findings may inform the design of future severe infection prevention RCTs in young infants in LMICs by (1) providing incidence estimates of various candidate case definitions and (2) supporting the planning of optimal outcome surveillance systems that balance the identification of severe infection cases with operational costs.

## Introduction

 Severe infections, including sepsis, contribute to a large burden of disease early in life and result in significant morbidity and mortality in young infants (<2 months).^1^,^3^ The burden of sepsis in young infants is particularly high in low and middle-income countries (LMICs).^4 5^ Randomised controlled trials (RCTs) of preventive and therapeutic interventions are essential for developing evidence-based guidelines for young infant management, but conducting such trials requires a case definition of severe infection relevant to this age group and the trial setting. Various definitions have been used to denote severe infection in young infants, including sepsis and serious bacterial infection (SBI). A major challenge limiting the comparability of RCTs of severe infection prevention and treatment interventions for young infants is the marked heterogeneity in definitions used in studies and guidelines.^6 7^ A positive sterile site culture is often regarded as the gold-standard definition of bacterial sepsis in infants, but these are often not available due to limited microbiological resources (blood culture bottles, lack of laboratory capacity, etc), inadequate sample volume, antibiotic administration prior to sample collection or low yield despite suitable sample collection without prior antibiotic administration.^8^ A systematic review of neonatal sepsis definitions used in RCTs identified a diverse range; the most common was culture-confirmed sepsis (27% of definitions), followed by clinical signs and culture confirmation (23%), and then clinical signs and laboratory investigation results (20%).^9^

In LMICs, sick young infants are often initially assessed in the community or at first-level health facilities where access to physicians and laboratory investigations is limited. In a cohort of young infants brought to a hospital or outpatient clinic for an acute illness, the Young Infants Clinical Signs Study Group developed a clinical sign-based algorithm (seven signs) to identify severe illnesses requiring urgent hospital management.^10^ The WHO has since adapted these seven signs to define clinical infection syndromes in young infants, including possible SBI (pSBI), which identify sick infants at risk of SBI or a severe illness requiring referral and/or empiric antibiotic treatment.^11 12^ The criteria have evolved over time, but currently, pSBI denotes one or more of the following: fever, hypothermia, poor feeding, history of convulsions, lethargy, elevated respiratory rate or severe chest indrawing.^11^

When designing RCTs for young infant severe infection prevention in LMICs, the use of a relatively permissive case definition (eg, pSBI) will result in higher event rates and, therefore, require a lower minimum sample size, compared with the use of more stringent case definitions (eg, culture-confirmed sepsis). However, the use of relatively permissive definitions may dilute intervention effects, meaning that experimental interventions that truly prevent bacterial infections may be misleadingly observed to have similar overall event rates as the control group. Conversely, when using a highly stringent severe infection case definition, the outcome may be so rare that the trial may be too costly or considered unfeasible to complete within a reasonable timeframe.

The Synbiotics for the Early Prevention of Severe Infections in Infants (SEPSiS) observational cohort study (NCT04012190) has several aims including the investigation of the incidence of severe infection in a cohort of young infants (0–60 days of age) born generally healthy in Dhaka, Bangladesh. The findings from this cohort were intended to be used to design severe infection prevention trials in generally healthy young infants. The primary case definition of severe infection selected for the SEPSiS study aimed to balance permissiveness and stringency while ensuring that the definition could be feasibly operationalised in the study setting. The case definition of severe infection combined physician documentation of standardised clinical signs and/or diagnosis of sepsis/SBI, plus either a positive sterile site culture or hospitalisation with an intention to treat with parenteral antibiotics for ≥5 consecutive days. The term *infection* was used rather than *sepsis* to acknowledge that many infants will not manifest overt signs of sepsis (ie, signs of systemic inflammatory response syndrome), and the qualifier *severe* implies that the infection may be life threatening or cause significant morbidity if untreated, such that a young infant would conventionally be admitted to the hospital and empirically treated with parenteral antibiotics.

We aimed to estimate the incidence of severe infection and non-injury-related death up to 60 days of age in Bangladeshi infants born generally healthy and to examine the effect of variations in severe infection case definitions on incidence estimates. We also examined the proportion of young infants with severe infection identified through various referral pathways as part of the study design. The results may inform the design of future severe infection prevention trials in young infants in LMICs and provide insight into optimal surveillance systems that balance the identification of cases with operational costs.

## Methods

### Study setting and participant eligibility

The study setting and eligibility criteria have been previously described.^13^ Between 25 November 2020 and 18 February 2022, potential mother–infant pairs were screened for eligibility at two government healthcare facilities in Dhaka city: Maternal and Child Health Training Institute in Azimpur and Mohammadpur Fertility Services and Training Centre. Additional details of study sites are provided in online supplemental section S1. Infants were eligible for inclusion if they were delivered at either of the two study hospitals, were 0–4 days of age (day of birth was defined as day 0), ≥1500 g at birth, orally feeding at the time of eligibility assessment, and the caregiver intended to maintain residence within the defined catchment area until the infant was 60 days of age. Infants were not eligible to participate if their birth weight was <1500 g, death or major surgery was considered highly probable during the first week of age, they had a major congenital anomaly of the gastrointestinal tract, they were receiving mechanical ventilation and/or cardiac support (eg, inotropes) and/or administration/prescription of parenteral antibiotics at the time of eligibility assessment, there was evidence of maternal HIV infection or prior antiretroviral treatment for presumed HIV infection, their mother had used a non-dietary probiotic supplement prenatally or in the postpartum period, they received postnatal administration of any non-dietary probiotic or prebiotic supplement, they were participating in a clinical trial involving administration of probiotics and/or prebiotics at the time of eligibility assessment, they were residing in the same household as a non-twin infant aged <60 days who was previously enrolled in this study or the other study that was implemented using the same research infrastructure (NCT05180201), or the infant was one of three or more liveborn infants from the same pregnancy. Additional details of eligibility criteria are shown in online supplemental section S2.

### Severe infection case definitions

#### Clinical severe infection and possible CSI used in SEPSiS community-based surveillance

##### SEPSiS clinical severe infection

≥1 of the following signs:

- Poor feeding (not sucking effectively or not sucking at all, on direct observation).
- Lethargy (movement only when stimulated or not moving at all, on direct observation).
- Convulsions (observed or strongly suspected by physician based on caregiver or community health research worker (CHRW) report).
- Severe chest in-drawing (observed).
- Fever (axillary temperature ≥37.5°C or rectal temperature ≥38°C).
- Hypothermia (axillary temperature <35.5°C or rectal temperature <36°C).

##### Possible SEPSiS clinical severe infection

Equivalent to SEPSiS CSI with the addition of fast breathing (measured respiratory rate ≥60 bpm) as a seventh sign.

### Primary definition of severe infection

At least one sign of SEPSiS clinical severe infection (CSI) documented by a study medical officer and/or non-study treating physician diagnosis of sepsis or another SBI; and at least one of the following two criteria:

- Non-study treating physician decision to admit to hospital, administration of ≥1 dose of a parenteral antibiotic on the day when SEPSiS CSI/sepsis/SBI is first ascertained, and treatment (or non-study treating physician intention to treat) with parenteral antibiotics for ≥5 consecutive days.
- Blood and/or cerebrospinal fluid (CSF) culture positive for a pathogenic bacterial or fungal organism.

Detailed explanations of the definition criteria are provided in online supplemental section S3. A committee with expertise in infectious diseases and microbiology developed a list of blood pathogens and contaminants *a priori* and refined it after the study was concluded based on the isolation of organisms that were not prespecified (online supplemental section S4).

### Variations in severe infection case definitions

WHO clinical infection syndromes include pSBI, CSI and critical illness.^11^

#### WHO possible SBI

≥1 sign of pSBI (poor feeding, convulsions, severe chest indrawing, fever (≥38°C), hypothermia (<35.5°C), lethargy, or fast breathing (≥60 breaths per minute in infants <7 days old)) documented by a study physician and non-study physician decision to admit to hospital.

#### Multiple signs of WHO pSBI

≥2 signs of pSBI documented by a study physician and non-study physician decision to admit to hospital.

#### WHO clinical severe infection

≥1 sign of CSI (poor feeding, severe chest indrawing, fever (≥38°C), hypothermia (<35.5°C), lethargy) documented by a study physician and non-study physician decision to admit to hospital. Note: WHO CSI (five signs) is similar to SEPSiS CSI (six signs) but excludes convulsions.

#### WHO critical illness

≥1 sign of ‘critical illness’ (poor feeding, convulsions or lethargy) documented by a study physician and non-study physician decision to admit to hospital.

#### Culture-confirmed severe infection

Blood and/or CSF culture positive for a pathogenic bacterial or fungal organism and non-study physician decision to admit to hospital.

#### Non-injury death

Any death that was not caused by an injury or accident per verbal autopsy.

A case definition was qualitatively considered to be *permissive* if it was expected to miss few cases of severe infection but might capture other diseases that were not severe bacterial infections (eg, viral infections). A case definition was considered relatively *stringent* if it was expected to capture few non-severe infection diseases but could miss cases of severe infection.

### Community-based surveillance

All data collected in this study were recorded by study personnel using an electronic data capture application run on handheld tablets. The criteria to meet the primary severe infection definition and alternative case definitions came from multiple study and/or non-study sources, but all of the criteria were entered into the electronic data capture application by study personnel.

Infants underwent surveillance for SEPSiS CSI from enrolment to 60 days of age. Up to 12 scheduled clinical assessments were performed by trained study medical officers on the day of enrolment, and by trained CHRWs 3 days postenrolment, 6 days postenrolment, and on days 10, 14, 21, 28, 35, 42, 49, 56 and 60 postnatal age. These assessments were conducted during in-person home or hospital visits or by telephone when an in-person visit was not feasible. At each scheduled visit, CHRWs assessed infants for signs of possible SEPSiS CSI. Infants with possible SEPSiS CSI ascertained by a CHRW were referred to the nearest study hospital for further assessment by a study medical officer. To identify illness episodes between scheduled visits, caregivers were asked to report any concerning signs to the study team via telephone. Infants with signs of possible SEPSiS CSI or other illness identified via an ad-hoc or follow-up assessment or phone call were referred to a study hospital for assessment by a study medical officer. Caregivers could also present directly to a study hospital to initiate an in-person visit with a study medical officer or a non-study treating physician, but also may have sought care at non-study facilities. The surveillance system to identify severe infection cases is shown in online supplemental figure S1. At enrolment, study nurses also collected information on maternal and infant demographics.

### SEPSiS CSI and sepsis/SBI case confirmation

When an infant with possible SEPSiS CSI presented to the study hospital, the study medical officer interviewed the caregiver and examined the infant. The infant was classified as having SEPSiS CSI if a study medical officer documented ≥1 sign of SEPSiS CSI. If the study medical officer documented SEPSiS CSI, the infant was referred to a non-study treating physician to confirm a clinical diagnosis of sepsis or SBI. A clinical diagnosis of sepsis was defined as sufficient general concern from the non-study treating physician to warrant a sepsis work-up (including blood culture). SBI was defined as an acute illness typically (or assumed to be) caused by bacteria. A list of infectious illnesses constituting SBI was established *a priori* and made available as a set of selectable response options for data entry (online supplemental section S5). The study medical officer selected the suitable diagnostic label based on the non-study treating physician’s diagnosis. Diagnoses could also be manually entered by study personnel using free text. These free-text diagnoses were adjudicated by three physician members of the SEPSiS team and classified as ‘likely SBI’, ‘possibly SBI’ or ‘not SBI’. Classification of manually entered ‘free text’ diagnoses is shown in online supplemental section S6.

If an infant was admitted to a non-study facility, study medical officers attempted to obtain clinical and laboratory investigation data as soon as possible. In these cases, documentation of clinical signs of SEPSiS CSI by study personnel was not possible. To determine a clinical diagnosis of sepsis or SBI, the study medical officer reviewed the treating physician diagnosis documented in the infant’s non-study facility medical record, extracted this information and aligned it with a standardised list of diagnostic labels.

### Sample collection and processing

A sepsis work-up was initiated if the infant was, or was intended to be, admitted to a hospital and had ≥1 sign of SEPSiS CSI present or had a diagnosis of sepsis and/or other SBI. Sepsis work-up laboratory analyses included complete blood count with differential, procalcitonin (PCT), high-sensitivity C reactive protein (CRP), alanine aminotransferase, glucose, direct and indirect bilirubin, creatinine, aerobic blood culture, urine dipstick, urine microscopy and culture, real-time RT-PCR on nasal swabs for influenza A and B, respiratory syncytial virus (RSV), ureaplasma and SARS-CoV-2 and skin swab for culture (where clinically indicated). If blood volume was lower than targeted (4.5 mL), assays were prioritised according to a predetermined sequence (online supplemental table S1). Although there was an intention to collect CSF where clinically indicated based on the non-study treating physician opinion, there were no instances in which CSF collection was performed.

For urine samples, clean catch or bagged urine collection was attempted. According to the operating procedures, if the urine dipstick was positive for leucocyte esterase and/or nitrites, a second sterile collection was to be attempted via bladder catheterisation, but this was never performed. Mid-turbinate nasal swabs were collected by using a single swab to obtain epithelial cells from both nostrils. For some suspected skin or soft tissue infections, a skin swab was collected by gently rotating a sterile swab over the infected area, or a pus sample was obtained by needle aspiration. Details on sample processing are provided in online supplemental section S7.

### Statistical analysis

A sample size of 2000 infants was estimated to obtain a desired precision of a 95% CI width of 2%, assuming a baseline severe infection incidence proportion of 5% and loss to follow-up of 5%. Participant baseline characteristics were summarised using descriptive statistics and stratified by complete and incomplete follow-up. Frequencies and percentages were reported for categorical variables. Means, SD, medians, 25th and 75th percentiles, minimum and maximum were computed for continuous variables. Incidence of severe infection, non-injury-related death and variations in severe infection case definitions were estimated using incidence rates and proportions. Incidence rates accounted for multiple incident episodes per infant while incidence proportions considered only the first severe infection episode for each infant. Incidence rates were calculated as the total number of severe infection episodes (using the primary and alternative severe infection definitions and accounting for multiple incident episodes per infant if these occurred) divided by the total number of infant-days at risk. Incidence proportions were calculated by plotting a Kaplan-Meier survival curve and subtracting the survival rate (proportion without the event) at 60 days from one. CIs were generated using an intercept-only Poisson regression with robust SEs to account for clustering within participants and a log offset of days at risk. Infants contributed person-time at risk if they were alive, had not voluntarily withdrawn from the study, were not hospitalised nor receiving parenteral antibiotics for a severe infection episode and had at least three severe infection-free days since the last day of administration of a prescribed parenteral antibiotic for an episode of severe infection or since the day of discharge from hospital (whichever was later). Infants were right-censored if they were lost to follow-up or died during the first 60 days of age. Loss to follow-up occurred if the infant missed the scheduled 3-month postnatal visit and the latest date of contact with study personnel was during the first 60 days of age. The date of loss to follow-up was defined as the latest date of contact with study personnel within the 60-day observation period postbirth. Infants who were lost to follow-up during the first 60 days of age contributed person-time at risk up to and including the latest date of contact with study personnel. Detailed definitions of at-risk and not-at-risk periods are provided in online supplemental section S8.

Proportions of severe infection cases identified from various pathways of the surveillance system were estimated. Laboratory investigation results and bacterial and viral aetiologies for cases of severe infection were also summarised.

Additional analyses explored the effect of variations in severe infection case definitions on incidence estimates within subgroups, including gestational age at enrolment (<37 weeks and ≥37 weeks), age at onset of severe infection (<28 days and 28–60 days) and sex. Sensitivity analyses for the calculation of the primary severe infection definition incidence proportions and rates were performed and included: (1) restricting to infants with ≥30 days of follow-up time; (2) varying the categorisation of manually entered free-text SBI diagnoses; (3) varying the duration of the severe infection-free period to determine re-entry into the subsequent at-risk period; (4) restricting the observation period to 0–59 days and (5) using the last scheduled date of contact with study personnel to define the date of loss to follow-up. Rationales for these analyses are described in online supplemental section S9.

Information on independent verification and code review for analyses is provided in online supplemental table S2. All statistical analyses were conducted using R V.4.2.3.

### Patient and public involvement

Caregivers/public were not involved in developing the research question, recruitment, design and conduct of the study or methods for study result dissemination.

## Results

**Figure 1:**
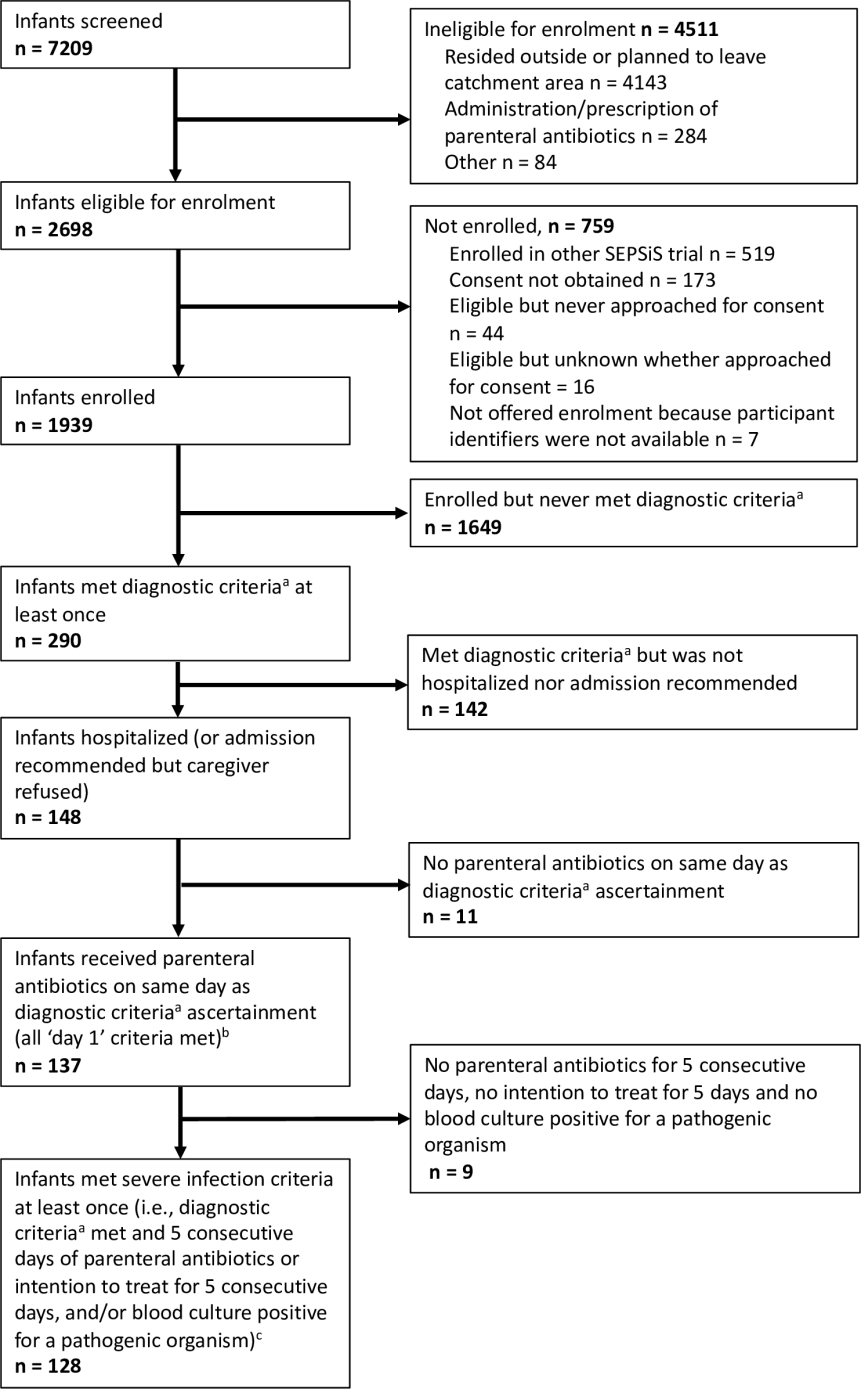
Study flow diagram. ^a^Diagnostic criteria: SEPSiS Clinical Severe Infection (CSI)/Sepsis/Serious Bacterial Infection (SBI) criteria were ascertained. ^b^Day 1 criteria: (1) Diagnostic criteria were ascertained; (2) there was administration of at least one dose of parenteral antibiotics; (3) the infant was admitted to hospital, already in hospital, or there was recommendation to admit the infant to hospital; (4) the infant’s age was less than or equal to 60 days. ^c^Among the 128 infants who met severe infection criteria at least once, there were 131 severe infection episodes (three infants had two severe infection episodes each during the observation period).

Of 1939 enrolled infants (figure 1 and table 1), 1522 (78%) had complete follow-up and 417 (22%) had incomplete follow-up (left catchment area at least once and/or lost to follow-up). There were no substantial differences in maternal and infant characteristics between infants who had complete follow-up compared with those who had incomplete follow-up (table 1).

**Table 1:** Participant characteristics, overall and by complete versus incomplete follow-up

Characteristic	Overall	Complete follow-up	Incomplete follow-up*
Number of infants, n (%)†	1939	1522 (78)	417 (22)
Maternal age (years), median (25th, 75th)	24 (20, 27)	24 (20, 28)	23 (20, 26)
Enrolment site, n (%)			
Maternal and Child Health Training Institute	651 (34)	522 (34)	129 (31)
Mohammadpur Fertility Services and Training Centre	1288 (66)	1000 (66)	288 (69)
Maternal education, n (%)‡			
None up to complete primary school§	540 (28)	427 (28)	113 (27)
Secondary incomplete	618 (32)	485 (32)	133 (32)
Secondary complete or higher	767 (40)	598 (40)	169 (41)
Parity, median (25th, 75th)	2 (1, 2)	2 (1, 2)	2 (1, 2)
First live birth, n (%)	827 (43)	625 (41)	202 (48)
Gestational age at delivery (weeks), median (25th, 75th)	39.1 (38.3, 40.1)	39.1 (38.3, 40.1)	39.1 (38.1, 40.1)
Term (≥37 weeks), n (%)	1769 (91)	1390 (91)	379 (91)
Preterm (<37 weeks), n (%)	150 (7.7)	118 (7.8)	32 (7.7)
Mode of delivery, n (%)			
Vaginal	875 (45)	675 (44)	200 (48)
C-section	1064 (55)	847 (56)	217 (52)
Maternal peripartum antibiotics administered, n (%)			
None	93 (4.8)	69 (4.5)	24 (5.8)
Intrapartum¶ only	97 (5.0)	78 (5.1)	19 (4.6)
Postpartum only	1425 (73)	1107 (73)	318 (76)
Both intrapartum¶ and postpartum	323 (17)	268 (18)	55 (13)
Sex, n (%)			
Male	926 (48)	730 (48)	196 (47)
Female	1013 (52)	792 (52)	221 (53)
Birth weight (g), mean (SD)	2872 (394)	2877 (396)	2854 (385)
Feeding pattern at or near enrolment**, n (%)			
Exclusively breastfed	1843 (95)	1453 (95)	390 (94)
Not exclusively breastfed or not breastfed	89 (4.6)	65 (4.3)	24 (5.8)
Infant age at enrolment (days), median (min, max)	1 (0, 4)	1 (0, 4)	1 (0, 4)
Scheduled home visits conducted per infant, median (min, max)	11 (0, 12)	11 (0, 12)	11 (0, 11)
In-person	9 (0, 12)	10 (0, 12)	7 (0, 11)
Telephone	1 (0, 11)	1 (0, 8)	2 (0, 11)
Infant age at exit from observation period, median (min, max)	60 (0, 60)	60 (5, 60)	60 (0, 60)
Follow-up time, median (25th, 75th)	59 (57, 59)	59 (58, 59)	50 (41, 55)

Variables with missing data were maternal age, maternal education, parity, gestational age at delivery, maternal peripartum antibiotics administered, and feeding pattern at or near enrolment. Missingness ranged from 0.05–1.03%.

* Incomplete follow-up refers to absence from the catchment area at least once n=378 (19%) and/or lost to follow-up n=42 (2.2%). Median duration of absence from catchment area (25th, 75th) in days is 6.0 (3.0, 11). Total number of days absent from catchment area for all infants was 4875 days. See *Statistical Analysis* section of Methods for details on how loss to follow-up was defined.

† The number of mothers for the 1939 infants is 1926.

‡ The denominator is the number of mothers.

§ Includes women with no education, incomplete primary school and completed primary school.

¶ Intrapartum period refers to antibiotics that were administered during labour and/or in the operating theatre, but prior to delivery.

** Mean age is 1.6 days.

**Figure 2:**
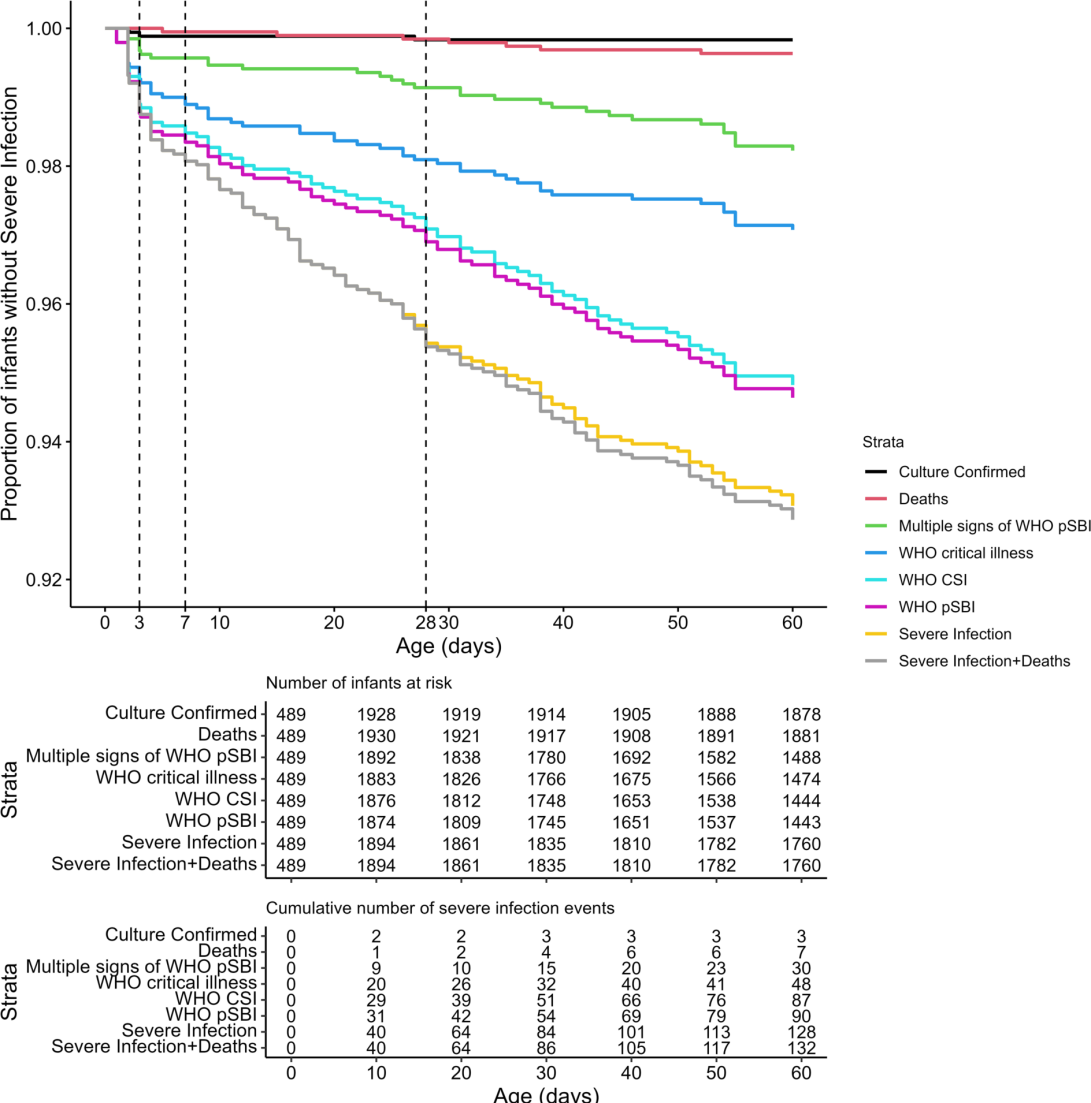
Kaplan-Meier curves for time to severe infection up to 60 days of age according to various severe infection case definitions. Number of infants at risk at time 0 (n=489) is lower than at 10 days because infants were recruited between days 0 and 4 after birth. CSI, clinical severe infection; pSBI, possible serious bacterial infection.

Using the primary severe infection definition, there were 131 severe infection episodes among 128 infants during the first 60 days of age (figure 1 and online supplemental figure S2). Three infants each had two incident severe infection episodes during the first 60 days of age (table 2). The severe infection incidence rate per 1000 infant-days at risk was 1.2 (0.97–1.4) using the primary definition and 0.84 (0.69–1.0) using WHO pSBI. Given that WHO CSI, WHO critical illness and multiple signs of WHO pSBI are subsets of WHO pSBI, they each had lower incidence rates than WHO pSBI (table 2 and online supplemental figure S3). The differences in incidence rates by case definition were maintained within subgroups including gestational age at enrolment (<37 weeks and ≥37 weeks), age at onset of severe infection (<28 days and 28–60 days of age) and sex (online supplemental tables S3–S5). Three infants had a severe infection episode with a positive blood culture for a pathogenic organism. Seven infants died from non-injury-related causes. Of these seven deaths, two infants had a severe infection, leading to their death. The incidence rate per 1000 infant-days at risk of blood culture-confirmed severe infection was 0.026 (0.0085-0.081), while that of non-injury death was 0.061 (0.029-0.13). The incidence rate of severe infection using the primary definition and non-injury deaths was 1.2 (1.0-1.4) per 1000 infant-days at risk. Time from birth to severe infection for the primary and alternative definitions is illustrated in figure 2.

**Table 2:** Incidence proportions and incidence rates of severe infection and variations in severe infection case definition (N_infants_=1939)

Severe infection case definition	Number of events	Number of infants with at least one event	Infant-days at risk	Incidence proportion*, per 1000 infants at risk (95% CI)	Incidence rate, per 1000 infant-days at risk (95% CI)
Severe infection	131	128	113 238	69 (58 to 81)	1.2 (0.97 to 1.4)
WHO possible serious bacterial infection†	92	90	109 316	54 (42 to 65)	0.84 (0.69 to 1.0)
WHO clinical severe infection‡	88	87	109 327	52 (41 to 63)	0.80 (0.65 to 0.99)
WHO critical illness§	48	48	109 677	29 (21 to 38)	0.44 (0.33 to 0.58)
Multiple signs of WHO possible serious bacterial infection	30	30	109 838	18 (11 to 24)	0.27 (0.19 to 0.39)
Culture-confirmed severe infection	3	3	114 335	1.7 (0.0 to 3.6)	0.026 (0.0085 to 0.081)
Death (non-injury)	7	7	114 384	3.6 (0.95 to 6.3)	0.061 (0.029 to 0.13)
Severe infection+deaths (non-injury)¶	136	132	113 238	69 (57 to 80)	1.2 (1.0 to 1.4)

* Estimated as 1 minus the survival probability at day 60. [Note: the analysis plan in the SEPSiS observational cohort study protocol also specified an additional method to calculate incidence proportions (dividing the number of severe infection events up to 60 days of age by the number of infants enrolled); however, we found the methods generated similar estimates, and we only reported the results using the Kaplan-Meier method because it accounts for right-censoring of infants (due to loss to follow-up or death) and was therefore considered to be less biased].

† At least one sign of possible serious bacterial infection (poor feeding, convulsions, severe chest indrawing, fever (≥38°C), hypothermia (<35.5°C), lethargy or fast breathing (≥60 breaths per minute in infants <7 days old)) documented by a study medical officer and non-study treating physician decision to admit to hospital.

‡ At least one sign of clinical severe infection (poor feeding, severe chest indrawing, fever (≥38°C), hypothermia (<35.5°C), or lethargy) documented by a study medical officer and non-study treating physician decision to admit to hospital.

§ At least one sign of critical illness (poor feeding, convulsions, or lethargy) documented by a study medical officer and non-study treating physician decision to admit to hospital.

¶ Not a cumulative sum of severe infection and deaths because two infants had severe infection and then died.

In all sensitivity analyses, there were no substantial differences in severe infection incidence proportions or rates using the primary definition (online supplemental table S6).

Of 131 primary definition severe infection episodes, the proportion with a completed sepsis work-up laboratory test ranged from 59% to 85%, depending on the test (online supplemental table S7). Of severe infection episodes for which sepsis work-up tests were done, the proportion of test results meeting their respective thresholds for clinical concern ranged from 4.8% for elevated ALT to 24% for elevated CRP. In the cohort, there were three positive blood cultures for a pathogenic organism, eight positive urine cultures for a pathogenic organism and four positive skin swab cultures taken from sites with suspected infections (online supplemental table S8). The primary severe infection definition captured all these events involving positive cultures, whereas the WHO pSBI definition only captured two of the three positive blood cultures, six of the eight positive urine cultures and none of the four positive skin swab cultures. Of primary severe infection definition episodes, 51% had an RT-PCR nasal swab, at least one of white blood cell count, neutrophil count, CRP or PCT, and a blood culture completed (online supplemental table S9). Of severe infection episodes for which these tests were done, 70% had a probable viral and/or bacterial infection (RT-PCR nasal swab positive for either RSV/influenza, evidence of a systemic inflammatory response or a positive urine dipstick or urine or blood culture). Most (67%) episodes were probable viral illnesses without bacterial infection (negative urine dipstick and bacterial cultures without evidence of a systemic inflammatory response or RSV/influenza positive). Only 36% were possible or confirmed bacterial infections without evidence of viral aetiology (evidence of a systemic inflammatory response and/or a positive urine dipstick or bacterial culture and RSV/influenza negative).

**Figure 3:**
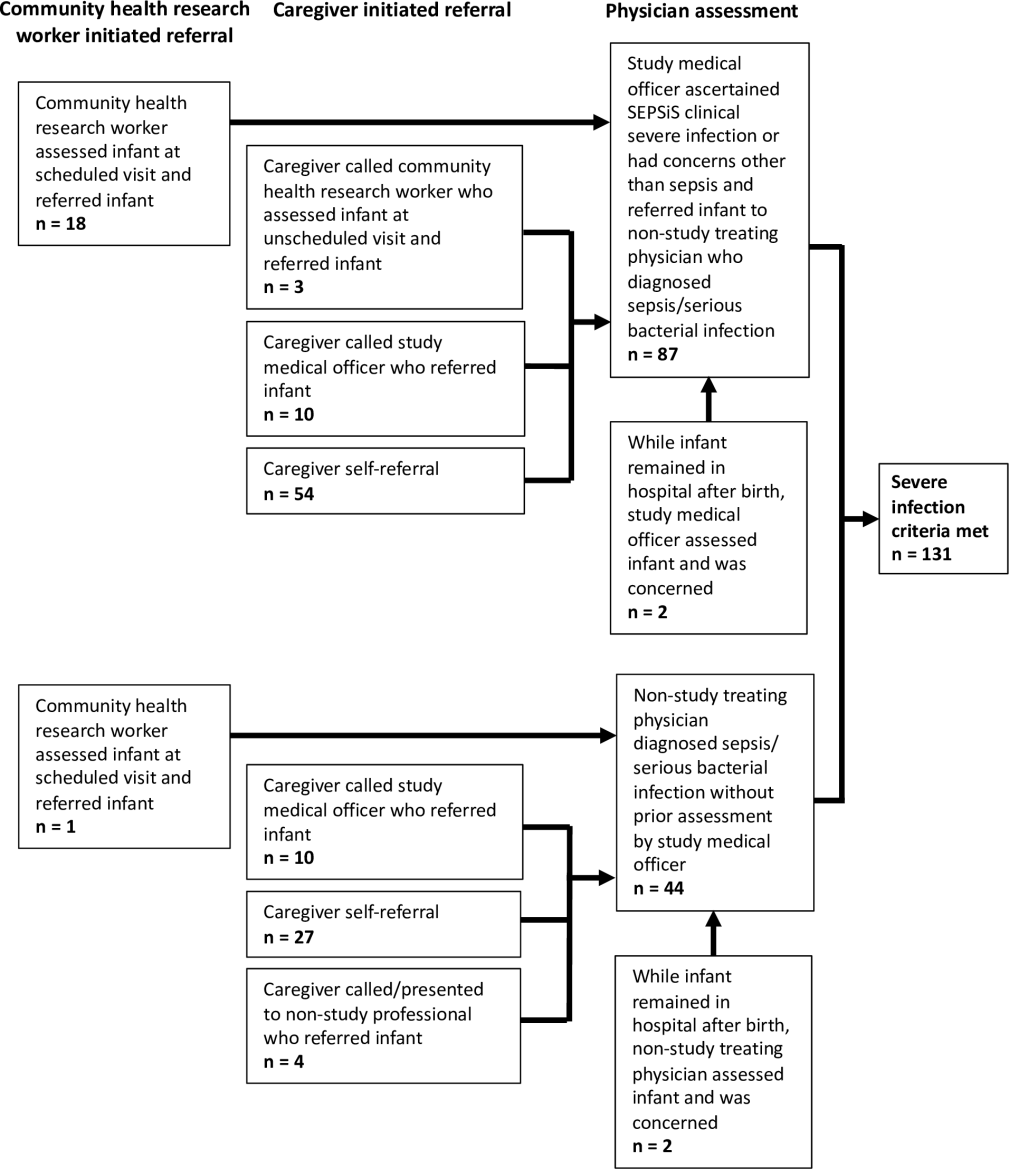
Referral pathways by which severe infection episodes were identified within the study. Infants may have had multiple assessments by study and non-study personnel for a severe infection illness episode prior to meeting final severe infection criteria. Frequencies are based on *initial presentation and assessment* for the severe infection episode.

Of the 131 severe infection episodes using the primary definition, 19 (15%) were identified directly following a scheduled CHRW home visit assessment and CHRW referral, and 112 (85%) were identified following caregiver self-referral (figure 3). For 43 (33%) severe infection episodes, the infants were assessed by a non-study treating physician without prior assessment by study personnel and met criteria for the primary severe infection definition through a non-study treating physician diagnosis of sepsis/SBI rather than documentation of clinical signs of SEPSiS CSI.

We conducted sample size calculations for a theoretical severe infection prevention RCT for young infants using various case definitions of severe infection (online supplemental table S10). The number of infants required per trial group is lowest using the primary severe infection definition (which had the highest incidence) and increases to very high sample sizes when using culture-confirmed infection.

## Discussion

The incidence of severe infection up to 60 days of age in Bangladeshi infants born generally healthy varied considerably by case definition. WHO clinical sign-based definitions such as pSBI yielded lower incidence rates than the primary severe infection definition, which comprised a more complex set of therapeutic and microbiologic criteria, including either the administration of ≥5 consecutive days of parenteral antibiotics and/or blood-culture confirmation. In practice, the primary definition was more permissive because the diagnostic component of the definition could be fulfilled by either a clinical sign documented by a study medical officer or a non-study treating physician diagnosis of sepsis or SBI, which enabled the definition to be applied to episodes for which a standardised assessment of clinical signs performed by study personnel was absent. Because many infants presented for care via self-referral to non-study physicians, definitions that required documentation of specific clinical signs by study personnel led to a substantial proportion of cases of non-study physician-diagnosed sepsis/SBI being missed.

The majority (85%) of severe infection cases using the primary definition were identified following caregiver self-referral, and it is plausible that many of the other cases identified following a CHRW scheduled visit may have also eventually been identified following caregiver self-referral, had the CHRW visit not been scheduled. In an urban setting such as Dhaka, health facilities are numerous and caregivers may thus seek care early for infant illness. Therefore, when conducting young infant severe infection prevention RCTs in such settings, frequent scheduled home assessments by study personnel to identify infants requiring referral may not be warranted, and resources may be better allocated towards other operational aspects of the RCT, such as staffing of study personnel at study hospitals and implementing mechanisms to retroactively identify cases that present to non-study physicians. However, in settings where caregivers have limited access to health facilities and may not readily seek care for infant illness (eg, some rural settings), or infants are receiving treatment including antibiotics at home, which may affect the yield of subsequent investigations (eg, blood culture), more frequent scheduled home assessments by study personnel to identify infants requiring referral may be warranted. It is also possible that frequent scheduled home visits could have facilitated earlier identification and management of mild infections, thereby preventing their progression to severe infections. This may affect incidence estimates and warrants consideration in RCT design.

In a previous observational study investigating the causes and incidence of community-acquired serious infections among infants 0–60 days of age in Bangladesh, India and Pakistan (ANISA), the incidence per 1000 live births was 95 for WHO pSBI and 1.6 for culture-confirmed bacterial infection.^14^ Notably, the SEPSiS observational study cohort was not a formal birth cohort and during the enrolment period from days 0 to 4 of age, infants were not eligible to be enrolled while receiving parenteral antibiotics. This likely led to the exclusion of cases of early-onset sepsis, which was intentional in the design of the SEPSiS study since its purpose was to guide the design of RCTs for severe infection prevention by postnatal interventions rather than treatment or prenatal or perinatal prevention. Therefore, it was expected that the incidence proportion of WHO pSBI in the SEPSiS cohort would be lower than in the ANISA cohort. However, the incidence proportion of culture-confirmed cases in the SEPSiS cohort was similar to the ANISA cohort. This similarity in the incidence of culture-confirmed cases was unexpected and may have been due to differences in blood culture processing techniques, organisms considered to be pathogenic and infectious disease specialist classification of isolates. In a cohort of facility-born infants in seven LMICs across South Asia and Africa, the incidence estimates of sepsis among infants 0–60 days of age were substantially higher than the estimates in both the SEPSiS and ANISA cohorts with 166 cases of clinically suspected sepsis per 1000 live births and 46.9 cases of blood culture-confirmed sepsis per 1000 live births.^4^ In addition to the SEPSiS cohort likely excluding many cases of early-onset sepsis, possible reasons for these discrepancies include differences in definition criteria and different *a priori* determination of pathogenic organisms and post hoc classification of isolates. These discrepancies further highlight that there may be substantial differences in incidence estimates of severe infection in young infants depending on the case definition used and source populations from which cases arise.

When formulating a case definition for young infant severe infection in LMICs, important considerations include balancing permissiveness and stringency and resources available to operationalise the case definition. The primary severe infection definition in the SEPSiS study was permissive by allowing for inclusion of cases that met clinical sign criteria and/or physician diagnosis of sepsis/SBI, but stringency was imposed by requiring administration of parenteral antibiotics for ≥5 days and/or blood culture confirmation. The criteria of treatment or intention to treat with parenteral antibiotics for ≥5 days objectively indicate a high level of clinical concern from a physician and have been used in case definitions in infant sepsis prevention RCTs.^15^ Compared with the WHO pSBI definition, the primary severe infection definition had higher sensitivity for capturing positive blood and urine cultures for pathogenic organisms. However, the laboratory investigation results of primary severe infection episodes suggest that this definition is likely still non-specific, since only about one-third of severe infection episodes were characterised as possible or confirmed bacterial infections based on laboratory criteria (ie, systemic inflammatory response and/or a positive urine dipstick or bacterial culture and RSV/influenza negative). While ~30% of episodes may have been non-infectious given they had no evidence of a systemic inflammatory response, were RSV/influenza negative and had negative urine dipstick and bacterial cultures; some of these episodes may have been caused by a non-RSV/influenza virus. The small proportion of blood culture-positive cases was partly due to challenges with adherence to testing protocols (eg, obtaining samples prior to antibiotic administration) but also because many cases meeting severe infection criteria were probably not bacterial infections.

International consensus criteria that incorporate laboratory investigation results, such as the Phoenix Sepsis Score, have been developed to identify sepsis in infants and children 0–18 years of age and are intended to be globally applicable.^16^ However, in the present study, the intended sepsis work-up laboratory investigations were only obtained in about half of severe infection episodes using the primary definition. Therefore, operationalisation of international consensus criteria such as the Phoenix Sepsis Score may not be feasible in similar LMIC settings. A consensus definition of severe infection in young infants that balances permissiveness and stringency and can be operationalised in LMICs would align research in this area and improve comparability of RCTs.

The SEPSiS observational cohort study was conducted over the course of the COVID-19 pandemic.^17^ In young infants, SARS-CoV-2 generally causes a mild viral illness without major acute complications^18 19^ and only one infant with severe infection using the primary definition was SARS-CoV-2 positive. Therefore, it is unlikely that SARS-CoV-2 directly affected incidence estimates of severe infection in this study. However, it is possible that the COVID-19 pandemic may have indirectly affected severe infection incidence estimates in both directions. For example, care-seeking behaviour may have decreased or been delayed during lockdown periods, leading to higher severe infection incidence.^20^ Conversely, social distancing measures may have decreased pathogen exposure, leading to lower severe infection incidence. It is difficult to predict the overall effect of the pandemic on young infant severe infection incidence estimates, but the pandemic is unlikely to have affected the differences in incidence estimates by case definition found in this study.

This study had several limitations. First, given that during the enrolment period from days 0 to 4 of age, infants were not eligible while receiving parenteral antibiotics, the incidence estimates of this study do not represent those of a birth cohort. However, the intention of this study was to inform the design of RCTs of severe infection prevention interventions in the postnatal period. Trialists can therefore use these findings to inform the selection of their case definition, sample size calculations and design of surveillance systems to identify cases. Second, the intended sepsis work-up laboratory investigations were only obtained in about half of severe infection cases using the primary definition, such that the inferences based on the laboratory investigation results may not be generalisable to all severe infection episodes. Third, the RT-PCR nasal swabs were limited to detection of RSV, influenza, SARS-CoV-2 and ureaplasma. More extensive microbiologic panels may have better characterised the causes of severe infection episodes. Finally, data collection by non-study personnel is limited by lack of standardisation. To mitigate the lack of standardisation, we employed expert panel adjudication. For example, free-text non-study physician diagnoses were adjudicated by three physician members of the study team and classified as likely, possibly or not SBI.

## Conclusion

In an observational cohort study in Dhaka, Bangladesh, the incidence of severe infection in young infants varied considerably by case definition. A severe infection definition that requires physician documentation of standardised clinical signs may miss a substantial proportion of cases identified by physician diagnosis of sepsis/SBI. A consensus definition of severe infection in young infants that balances permissiveness and stringency and can be operationalised in LMICs would improve the comparability of RCTs. If health facilities are accessible and caregivers readily seek care for infant illness, frequently scheduled home assessments by study personnel to identify infants requiring referral may not be warranted.

## Supplementary material

10.1136/bmjph-2024-002383 online supplemental file 1

## Data Availability

Data are available in a public, open access repository.
